# A new machine-learning method to prognosticate paraquat poisoned patients by combining coagulation, liver, and kidney indices

**DOI:** 10.1371/journal.pone.0186427

**Published:** 2017-10-19

**Authors:** Lufeng Hu, Huaizhong Li, Zhennao Cai, Feiyan Lin, Guangliang Hong, Huiling Chen, Zhongqiu Lu

**Affiliations:** 1 Department of Pharmacy, The First Affiliated Hospital of Wenzhou Medical University, Wenzhou, China; 2 Department of Computing, Lishui University, Lishui, Zhejiang, P. R. China; 3 College of Physics and Electronic Information Engineering, Wenzhou University, Wenzhou, China; 4 Department of Emergency, The First Affiliated Hospital of Wenzhou Medical University, Wenzhou, China; University of Virginia, UNITED STATES

## Abstract

The prognosis of paraquat (PQ) poisoning is highly correlated to plasma PQ concentration, which has been identified as the most important index in PQ poisoning. This study investigated the predictive value of coagulation, liver, and kidney indices in prognosticating PQ-poisoning patients, when aligned with plasma PQ concentrations. Coagulation, liver, and kidney indices were first analyzed by variance analysis, receiver operating characteristic curves, and Fisher discriminant analysis. Then, a new, intelligent, machine learning-based system was established to effectively provide prognostic analysis of PQ-poisoning patients based on a combination of the aforementioned indices. In the proposed system, an enhanced extreme learning machine wrapped with a grey wolf-optimization strategy was developed to predict the risk status from a pool of 103 patients (56 males and 47 females); of these, 52 subjects were deceased and 51 alive. The proposed method was rigorously evaluated against this real-life dataset, in terms of accuracy, Matthews correlation coefficients, sensitivity, and specificity. Additionally, the feature selection was investigated to identify correlating factors for risk status. The results demonstrated that there were significant differences in the coagulation, liver, and kidney indices between deceased and surviving subjects (*p*<0.05). Aspartate aminotransferase, prothrombin time, prothrombin activity, total bilirubin, direct bilirubin, indirect bilirubin, alanine aminotransferase, urea nitrogen, and creatinine were the most highly correlated indices in PQ poisoning and showed statistical significance (*p*<0.05) in predicting PQ-poisoning prognoses. According to the feature selection, the most important correlated indices were found to be associated with aspartate aminotransferase, the aspartate aminotransferase to alanine ratio, creatinine, prothrombin time, and prothrombin activity. The method proposed here showed excellent results that were better than that produced based on blood-PQ concentration alone. These promising results indicated that the combination of these indices can provide a new avenue for prognosticating the outcome of PQ poisoning.

## 1. Introduction

Paraquat (1,1′-dimethyl-4,4′-bipyridium dichloride, PQ) has been one of the most widely used, nonselective, contact herbicides since it was first manufactured in 1962 [1]. Although it is safe in agricultural activities, PQ is more toxic to humans than any other herbicidal agent and has caused numerous deaths worldwide [2].After ingestion, PQ is rapidly absorbed and distributed to lung, liver, kidney, and muscle, with a volume of distribution at1.2–1.6 L/kg [3]. If not treated in time, PQ accumulation in the body leads to fulminant organ failure, including pulmonary edema, cardiac, kidney, and hepatic failure [4]. Therefore, confirming the PQ-poisoning diagnosis and risk assessment in a timely manner is particularly important.

To date, many studies have shown that PQ concentrations, especially plasma PQ concentrations, are highly correlated to the prognosis of PQ poisoning, with the measurement of plasma and urine PQ concentrations confirmed as the most useful test in PQ poisoning [5–7]. Although other laboratory examinations, such as coagulation function, hepatic and kidney function tests have been recommended as useful in evaluating PQ poisoning, their prognostic significance has remained unclear. Moreover, these laboratory examinations have included many indices whose clinical significance resists identification.

In previous studies here, the degree of PQ toxicity and diagnosed PQ poisoning inpatients by blood routine tests (BRT) were investigated as well as complete blood counts and arterial blood gas analyses [8, 9]. An intelligent machine learning-based method was developed to predict PQ poisoning risk status. However, two laboratory examinations did not achieve higher predictive ability than PQ plasma concentrations. In this study, an attempt was made to predict the prognosis of PQ poisoning by combining coagulation, liver, and kidney indices in a new, improved, intelligent machine learning method based on an extreme learning machine (ELM) [10]. ELM is a new learning algorithm for a single hidden layer, feed-forward, neural network. Different from the common parameter tuning strategy of neural networks, ELM attempts to choose input weights and hidden biases randomly, and the output weights are analytically determined using the Moore-Penrose generalized inverse. ELM not only learns much faster with higher generalization performance but also keeps very few parameters for tuning. Thanks to its desirable properties, ELM has been applied in various of fields, such as disease diagnosis [11–13], overweight detection [14], image quality assessment [15], facial recognition [16–18], micro-expression recognition [17], land cover classification [19], anomaly detection in traffic [20], biofuel engine performance prediction [21], and hyperspectral images classification [22].

The prognostic accuracy in PQ poisoning assessment was further improved by proposing an effective feature selection method based on grey wolf optimization (GWO) [23], which is a new metaheuristic that has shown excellent performance in other optimization problems [24–31]. In this paper, GWO was first proposed to combine with the effective ELM classifier to identify the most significant indices correlated with risk status. The resultant model, a GWO-ELM, was examined in terms of classification accuracy, Matthews Correlation Coefficients (MCC), sensitivity, and specificity on real data samples collected from The First Affiliated Hospital of Wenzhou Medical University. For comparison purposes, other metaheuristics, including particle swarm optimization (PSO) and genetic algorithm (GA), also based on ELM were performed on the same data. Promisingly, the developed GWO-ELM method achieved the highest prediction accuracy, MCC, sensitivity, and specificity of 86.55%, 0.7407, 81.24%, and 90.48%, respectively.

The remainder of this paper was organized as follows. Section 2 presents details of patients and data, Section 3 details the proposed prediction model, and Section 4 details the experimental designs. Section 5 includes experimental results from the proposed method, Section 6 offers a discussion of experimental results, and Section 7 summarizes the conclusions and recommendations for future work.

## 2. Patients

### 2.1 Ethics statement

There were 103 PQ-poisoning patients admitted to the First Affiliated Hospital of Wenzhou Medical University from January 1, 2013, to December30, 2015, who had positive contact history and diagnosed as PQ poisoning patients were inducted into the study. Information from PQ poisoning patients that could identify individual participants were assessed and handled by the authors during data collection. All clinical examination and data collection of PQ patients were conducted in accordance with the Declaration of Helsinki and approved by the Medical Ethics Committee of the First Affiliated Hospital of Wenzhou Medical University (Register number: 2016153).

### 2.2 Sample preparation

All PQ poisoning patients included in this study arrived at the emergency room (ER) within 24 h of the poisoning incident and did not receive any invasive treatment, such as blood transfusion and any intravenous drug treatments. All selected PQ-poisoning patients received the same treatment plans [32]. They were divided into two groups, deceased group and survival groups according to their treatment outcomes, which were assessed by vital signs, such as respiratory rate, heart rate, and blood pressure.

Patient blood samples were collected as follows. When PQ patients arrived in the ER, 2mL of blood was collected into heparin lithium-anticoagulant tubes (purple tube) for coagulation, liver, and kidney tests. Another 2mL blood was collected for determination of PQ plasma concentration. Coagulation, liver, and kidney tests were determined assessed using an AU 5800 Beckman automated clinical chemistry analyzer (Beckman Coulter, Inc., Brea, CA, USA). PQ plasma concentrations were determined by high performance liquid chromatography (HPLC) conducted in an Agilent 1260 Infinity HPLC system 1260 (Agilent Technologies, Inc., Santa Clara, CA, USA). The HPLC analysis has been validated and successfully applied in PQ determination, as described in our previously published study [32].

## 3. Methods description

**Fig 1:**
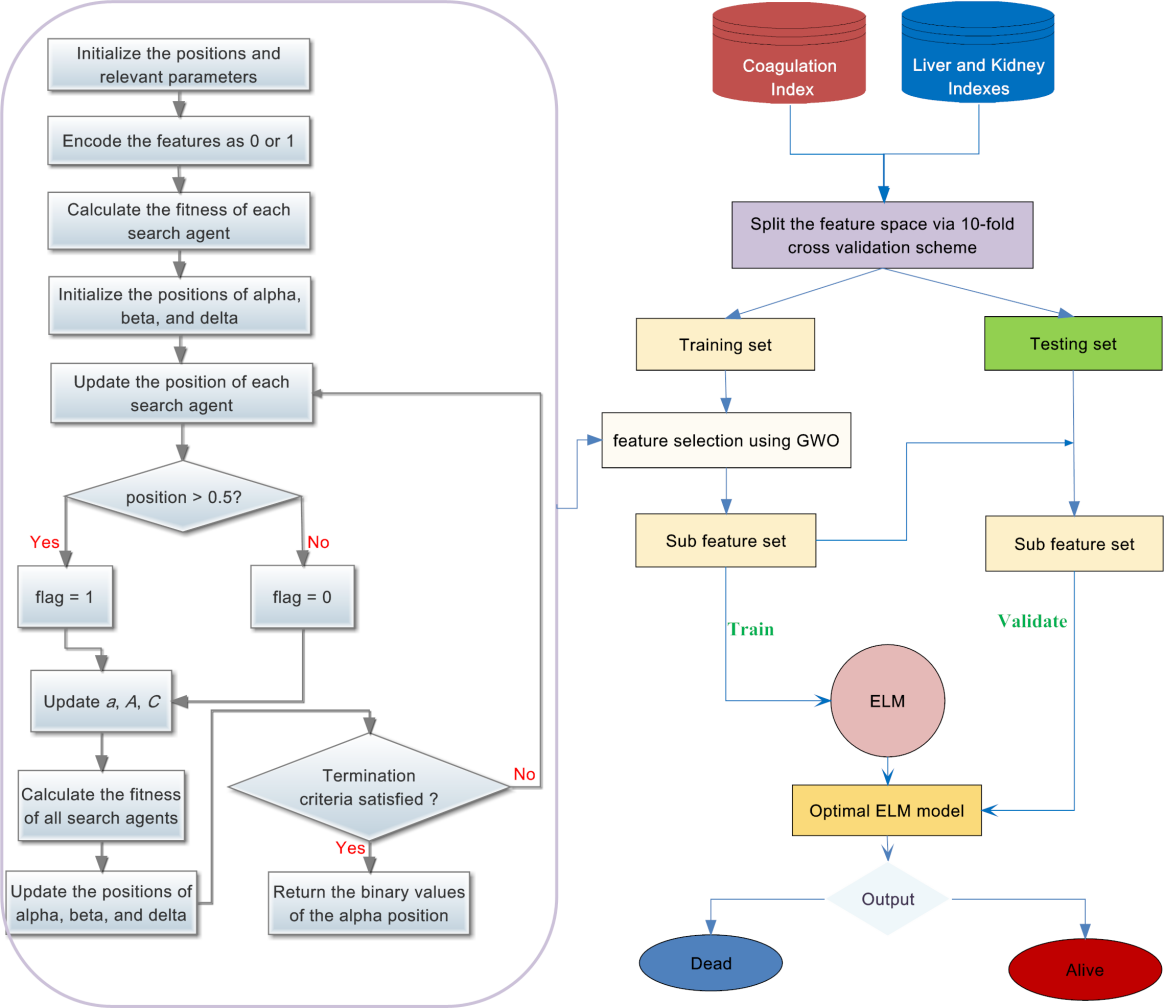
Flowchart of the proposed method.

In this section, the proposed GWO-ELM prediction method for PQ prognosis is described in detail. The main procedure of the GWO-ELM method is shown in Fig 1. As shown, the input was a combination of coagulation, liver, and kidney indices, and then the data were split into a training set and testing set via a 10-fold cross-validation strategy. GWO was wrapped with a fast and effective ELM classifier to perform the feature subset selection on the training set. After the optimal feature subset was obtained, the optimal ELM was constructed. Then, the optimal model was employed to conduct prognosis determination son the testing set with the same feature subset obtained in the training stage. The final output of the predictive model showed two statuses, ‘1’ means the PQ patient was deceased, while ‘0’ represents the patient as alive.

The detailed procedure of the proposed GWO-ELM method is presented below.

- Step 1: Initialize the input parameters for GWO, including population size, maximum number of iterations, upper bound of variables, lower bound of variables, and the dimension of the problem.
- Step 2: Initialize a population of grey wolves randomly based on the upper and lower bounds of the variables.
- Step 3: Initialize *a*, $\vec{A}$, and $\vec{C}$ using the following equations.
  α=2 (1)
  $$\vec{A}=2a\cdot {\vec{r}}_{1}-a$$ (2)
  $$\vec{C}=2{\vec{r}}_{2}$$ (3)
  where ${\vec{r}}_{1}$ and ${\vec{r}}_{2}$ are random vectors in [0, 1].
- Step 4: Encode each grey wolf with *n* dimensions. Then, the *n* dimensions are discredited to binary values by Eq (4), in which ‘1’ indicates the feature selected and ‘0’ indicates the feature not selected.
  $$flag_{i,j}=\left\{ \begin{array}{l} 1\;X_{i,j}>0.5\\ 0\;otherwise \end{array}\right.$$ (4)
  where *X*_*i*,*j*_ indicates the *j*th position of the *i*th grey wolf.
- Step 5: Calculate the fitness with the selected features for each grey wolf according to the following equation.
  $$\left\{ \begin{array}{l} f_{1}=\frac{\sum_{i=1}^{K}acc_{i}}{K}\\ f_{2}=1-\frac{\sum_{j=1}^{n}bin_{j}}{n}\\ f=w_{1}\times f_{1}+w_{2}\times f_{2} \end{array}\right.$$ (5)
  The first sub-objective function *f*_*1*_ represents the average accuracy achieved by the ELM classifier via *K*-fold cross validation (CV), where *K* = 5 and *acc*_*i*_ is the accuracy of the *i*th fold CV. In the second sub-objective function *f*_*2*_, *bin*_*j*_ is the binary value of the *j*th feature and *n* the total number of features. In objective function *f*, *w*_1_ is the weight for ELM classification accuracy and *w*_2_the weight for the selected features.
- Step 6: Select the first three best grey wolves that have maximum fitness and save them as *α*, *β*, and *δ*, respectively.
- Step 7: Update the position of the rest of the population (*ω*) using Eqs (6–12).
  $${\vec{D}}_{\alpha }=\left|{\vec{C}}_{1}\cdot {\vec{X}}_{\alpha }-\vec{X}\right|$$ (6)
  $${\vec{D}}_{\beta }=\left|{\vec{C}}_{2}\cdot {\vec{X}}_{\beta }-\vec{X}\right|$$ (7)
  $${\vec{D}}_{\delta }=\left|{\vec{C}}_{3}\cdot {\vec{X}}_{\delta }-\vec{X}\right|$$ (8)
  $${\vec{X}}_{1}={\vec{X}}_{\alpha }-{\vec{A}}_{1}\cdot {\vec{D}}_{\alpha }$$ (9)
  $${\vec{X}}_{2}={\vec{X}}_{\beta }-{\vec{A}}_{2}\cdot {\vec{D}}_{\beta }$$ (10)
  $${\vec{X}}_{3}={\vec{X}}_{\delta }-{\vec{A}}_{3}\cdot {\vec{D}}_{\delta }$$ (11)
  $$\vec{X}(t+1)=\frac{{\vec{X}}_{1}+{\vec{X}}_{2}+{\vec{X}}_{3}}{3}$$ (12)
  Where *t* is the current iteration
- Step 8: Return back if the grey wolves go beyond the boundaries of the variables.
- Step 9: Decrease the value of *a* from 2 to 0 linearly.
- Step 10: Update $\vec{A}$ and $\vec{C}$ using the Eqs (2) and (3), respectively.
- Step 11: Go to step 4 if the stopping criterion is not satisfied.
- Step 12: Return the *n* dimensions’ binary values of *X*_*α*_ as markers of the best feature subset.

### 3.1 Grey Wolf Optimization (GWO)

The GWO is a new swarm intelligence algorithm proposed by Mirjalili et al. [23], which is based on a hunting mechanism and the social hierarchy of grey wolves in nature. It is comprised of three main steps, including hunting for prey, encircling prey, and attacking prey. In GWO, the best solution is assumed as alpha, and the second and third best solutions coined as beta and delta, respectively. The rest of the candidate solutions are assumed to be omega. The hunt is usually guided by the alpha wolf. The beta and delta wolves might also occasionally participate in hunting. In an abstract search space, the prey's location, however, is unknown. The mathematical hunting behavior of the alpha wolf and other wolves are mimicked by supposing that the beta and delta wolves possess better knowledge regarding the prey's potential location. The first three best solutions obtained at this point are therefore saved and the other search agents obliged to update their positions.

To search in a discrete space, the following equation is introduced to update the grey wolf positions.

$$flag_{i,j}=\left\{ \begin{array}{l} 1\;X_{i,j}>0.5\\ 0\;otherwise \end{array}\right.$$ (13)

Where *X*_*i*,*j*_ indicates the *j*th position of the *i*th grey wolf.

### 3.2 Extreme Learning Machine (ELM)

The traditional feed-forward neural network, using an iterative gradient descent algorithm to adjust weight parameters, has obvious defects, including 1) slow learning speed, 2) difficulty in determining the learning rate and can easily to fall into a local minimum, and 3) prone to overtraining, causing extensive performance decline. These defects become constrained by the widespread use of iterative algorithms for feed-forward neural networks. To solve these problems, Huang et al. have proposed an extreme learning machine (ELM) [33, 34], based on the Moore-Penrose (MP) generalized inverse matrix theory, which has led to the minimum norm least-squares (LS) solution. This solution is unique and has the smallest norm among all the LS solutions. Compared with the iterative algorithm, ELM greatly improves the network generalization ability and learning speed, as it does not need to adjust the weights of input and hidden layers.

In summary, the learning steps of the ELM algorithm can be summarized as the following three steps:

1. Randomly assign input weights and bias,
2. Calculate the hidden layer output matrix, and
3. Calculate the output weight.

## 4. Experimental designs

### 4.1 Statistical analysis

First, the differences in the coagulation, liver, kidney, and glucose indices between deceased group and survival groups were analyzed by the Independent-Sample Test. Then, the prognostic values of coagulation, liver, kidney and glucose results in PQ poisoning were analyzed using a receiver-operating characteristic (ROC) curve. As plasma PQ concentration has been identified as the index most associated with PQ poisoning, it was also analyzed by ROC curve and compared with these biochemical indices. The sensitivity and specificity of the machine learning method was evaluated using the Fisher discriminant to classify the deceased and survival groups that performed at stepwise method using Wilks' lambda option. All these statistical analyses were performed using the software SPSS version 21.0 (IBM SPSS, Armonk, NY, USA). The level of significance was set at *p*<0.05.

### 4.2 Machine learning

The proposed prognostic system was implemented using MATLAB. The implementation code, available at http://www3.ntu.edu.sg/home/egbhuang, was used to construct the ELM model. GWO, PSO, and GA were implemented from scratch. The empirical experiment was conducted on a AMD Athlon 64 X2 Dual Core Processor 5000+ (2.6 GHz) with 4 Gb of RAM and operating system Windows 7.

Data were scaled into the range [–1, 1] before classification. The stratified 10-fold CV [35] was used to evaluate classification performance to guarantee unbiased results. The number of the maximum iterations and swarm size were set at100 and 25, respectively. Commonly used evaluation criteria, such as classification accuracy (ACC), sensitivity, specificity, and MCC, were used to evaluate the proposed method. They are defined as follows.

$$ACC=\frac{TP+TN}{TP+FP+FN+TN}\times 100\%$$ (14)

$$Sensitivity=\frac{TP}{TP+FN}\times 100\%$$ (15)

$$Specificity=\frac{TN}{FP+TN}\times 100\%$$ (16)

$$MCC=\frac{TP*TN-FP*FN}{\sqrt{\left(TP+FP)*(TP+FN)*(TN+FP)*(TN+FN\right)}}\times 100\%$$ (17)

Where TP is the number of true positives, which indicated cases wherein the “deceased” class was correctly predicted; FN the number of false negatives, which indicated cases wherein the “deceased” class was incorrectly predicted; TN the number of true negatives, which indicated cases wherein the “survival” class was correctly predicted; and FP the number of false positives, which indicated cases where in the “survival” class was incorrectly predicted.

## 5. Results

### 5.1 Data collection

A total of 103 PQ-poisoned patients who met the required criteria (male 56, female 47) were included. The first time tests for coagulation, liver, kidney indices and PQ plasma concentrations of the two groups, with the patients’ ER arrival without any treatment, were recorded. In addition, the last blood tests for coagulation, liver, and kidney indices after the patients received a series treatment were also recorded. There was a total of 20 blood indices included in this study(Table 1). The coagulation indices included PT, PTA, INR, FIB, ATPP, RAPTT, TT, and PTR; liver indices included TBil, DBIL, IBIL, TP, Alb, Alb/Glo, ALT, AST, ALT/AST, and Glu; kidney indices included BUN and CR. The first time tests of coagulation, liver, kidney indices were listed in S1 Dataset. PQ concentrations in the survival and deceased groups were725.46±996.43 ng/mL and 59092.84±123688.59ng/mL, respectively.

**Table 1:** The indices of coagulation, liver, kidney test in this study.

No.	Index	Abbreviation
F_1_	prothrombin time	PT
F_2_	prothrombin activity	PTA
F_3_	international normalized ratio	INR
F_4_	Fibrinogen	FIB
F_5_	activated partial thromboplastin	ATPP
F_6_	ratio of APTT	RAPTT
F_7_	thrombin time	TT
F_8_	prothrombin time ratio	PTR
F_9_	total bilirubin	TBil
F_10_	direct bilirubin	DBIL
F_11_	indirect bilirubin	IBIL
F_12_	total protein	TP
F_13_	albumin	Alb
F_14_	albumin-globulin ratio	Alb/Glo
F_15_	alanine aminotransferase	ALT
F_16_	aspartate aminotransferase	AST
F_17_	ratio of aspartate aminotransferase to alanine	ALT/AST
F_18_	Glucose	Glu
F_19_	urea nitrogen	BUN
F_20_	creatinine	CR

### 5.2 Differences between biochemical indices

The first and last coagulation, liver, and kidney tests for the selected patients showed that there were clearly differences between the two groups (Table 2). In first time test, there were two coagulation indices PT and PTA, six liver indices TBil, DBIL, IBIL, ALT, AST and ALT/AST, tow kidney indices BUN and CR had statistical differences (*p*<0.05), especially, PTA, TBil, DBIL, and AST had significant difference (*p*<0.001). In last time test, three coagulation indices PT, PTA and FIB, eight liver indices TT, TBil, DBIL, IBIL, TP, Alb, ALT and AST, tow kidney indices BUN and CR showed statistical differences *(p<*0.05). Compared with the first time tests, the changed indices of PT, PTA, TBil, DBIL, IBIL, ALT, AST, BUN, and CR still exhibited statistically significant differences(*p*<0.05) after the PQ patients received a series of treatments. This indicated that the influence (or damage) of PQ poisoning on these indices was persistent and these indices correlated greatly with PQ poisoning. However, among these changed indices, which index possessed important clinical meaning in predicting the prognosis of PQ poisoning remained unclear.

**Table 2:** Variance analysis of PQ poisoned patients in deceased group (n = 52) and survival group (n = 51).

Index	First time test			Last time test		
	Survival	Deceased	P	Survival	Deceased	P
PT	15.14±3.42	17.35±4.39	0.006	13.70±2.63	17.19±4.73	<0.001
PTA	81.72±12.01	65.29±21.45	<0.001	88.21±13.54	69.52±26.11	<0.001
INR	1.16±0.13	5.08±25.51	0.280	1.10±0.11	3.05±10.41	0.238
FIB	2.77±1.35	6.25±25.35	0.334	2.93±1.28	4.40±1.90	<0.001
ATPP	89.89±61.38	100.57±63.59	0.397	47.21±37.73	65.23±40.44	0.061
RAPTT	1.71±1.08	1.83±1.12	0.649	1.04±0.24	3.34±10.71	0.195
TT	98.27±76.25	111.05±79.94	0.440	40.14±52.46	81.37±74.69	0.013
PTR	2.69±2.89	2.12±3.77	0.538	1.70±2.05	9.34±34.91	0.276
TBil	18.48±23.91	41.08±33.22	<0.001	16.63±33.18	141.35±160.54	0.001
DBIL	9.40±19.29	28.98±29.61	<0.001	10.00±26.67	115.65±132.33	0.001
IBIL	9.45±5.49	12.32±5.55	0.013	7.53±7.45	25.70±31.63	0.013
TP	64.83±6.07	64.47±6.50	0.777	60.53±6.28	55.01±10.11	0.024
Alb	38.14±4.39	37.86±4.72	0.769	34.11±3.56	30.07±4.68	0.001
Alb/Glo	1.45±0.26	1.45±0.25	0.936	1.32±0.19	1.26±0.27	0.348
ALT	63.82±111.80	168.94±183.42	0.001	72.45±99.32	293.35±292.40	0.002
AST	47.10±58.27	240.38±225.84	<0.001	37.43±63.24	161.78±147.43	0.001
ALT/AST	1.05±0.73	0.67±0.40	0.002	2.12 ±1.00	2.19±1.88	0.875
Glu	7.65±2.27	8.54±3.99	0.178	5.56±1.51	6.24±2.01	0.111
BUN	5.53±4.63	7.69±5.31	0.030	7.38±3.58	13.40±6.86	<0.001
CR	95.02±109.59	167.71±115.86	0.001	97.55±74.18	216.03±111.86	<0.001

### 5.3 Prognostic values analysis

**Fig 2:**
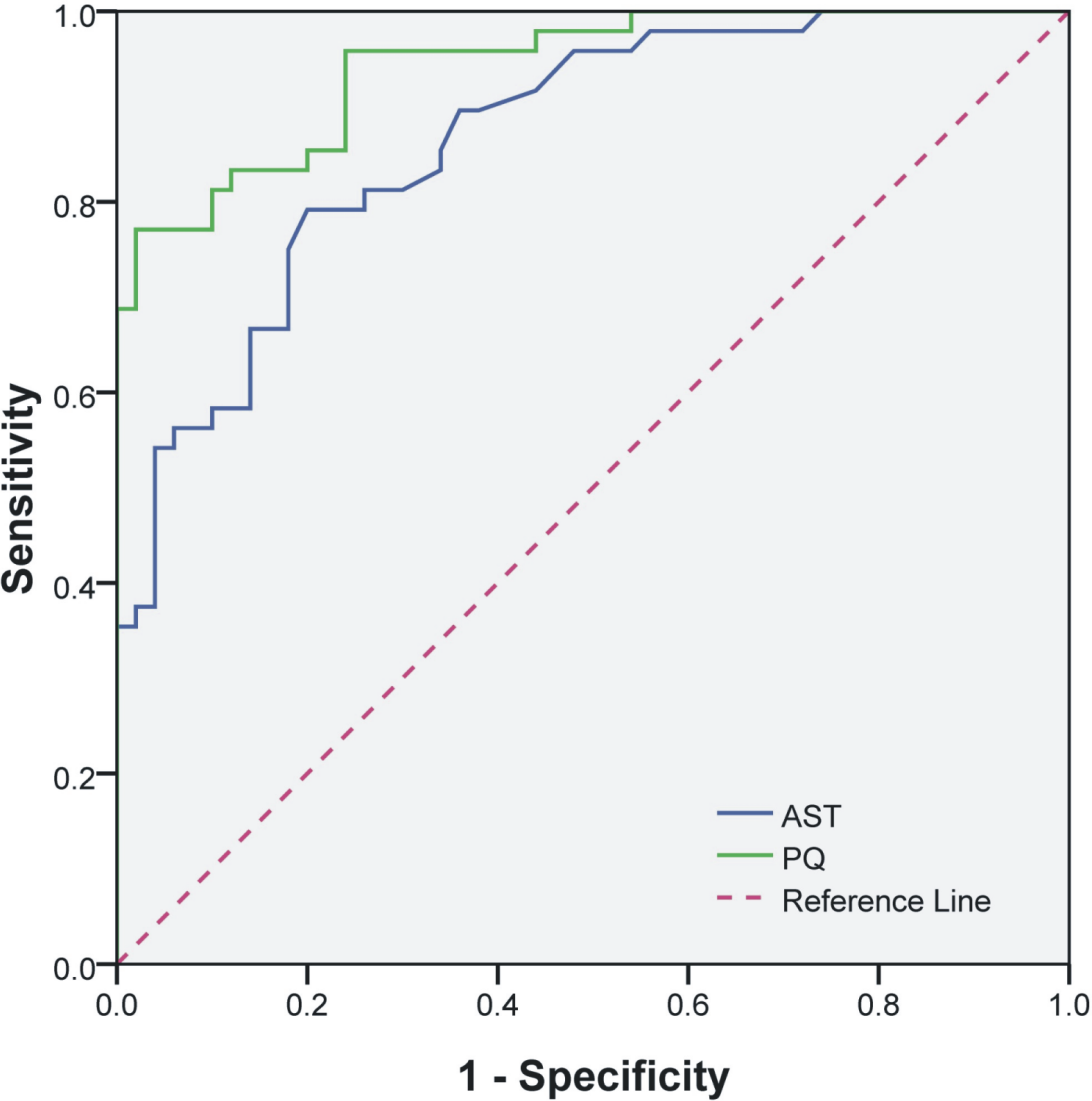
Receiver-operating characteristic (ROC) curves for PQ concentration and AST level in PQ-poisoned patients.

Prognostic values were evaluated by carrying out ROC curve and Fisher discriminant analyses. The ROC curve analysis showed that PT, PTA, INR, TBil, DBIL, IBIL, ALT, AST, ALT/AST, BUN, and CR showed statistical significance (*p*<0.05) in predicting the prognosis of PQ poisoning, which was consistent with the result of variance analysis. Among these indices, AST exhibited the highest area under the receiver-operating characteristic curve, which was close to the PQ concentration (Fig 2). The results of ROC analysis are listed in Table 3. Fisher discriminant analysis showed that 74.5% of original grouped cases were correctly classified when all indices of coagulation, liver, and kidney were selected as the independent variables.

**Table 3:** ROC curves analysis of PQ concentration and coagulation, liver and kidney indices.

Variable	AUC	95% Confidence Interval		P
		Lower	Upper	
PQ	0.933	0.885	0.981	<0.001
PT	0.685	0.573	0.796	0.002
PTA	0.298	0.19	0.406	0.001
INR	0.699	0.59	0.808	0.001
TBil	0.805	0.711	0.9	<0.001
DBIL	0.800	0.705	0.895	<0.001
IBIL	0.698	0.588	0.807	0.001
AST	0.851	0.775	0.927	<0.001
ALT	0.722	0.618	0.826	<0.001
ALT/AST	0.337	0.226	0.447	0.007
BUN	0.706	0.597	0.815	0.001
CR	0.784	0.69	0.878	<0.001

### 5.4 ELM classification

**Fig 3:**
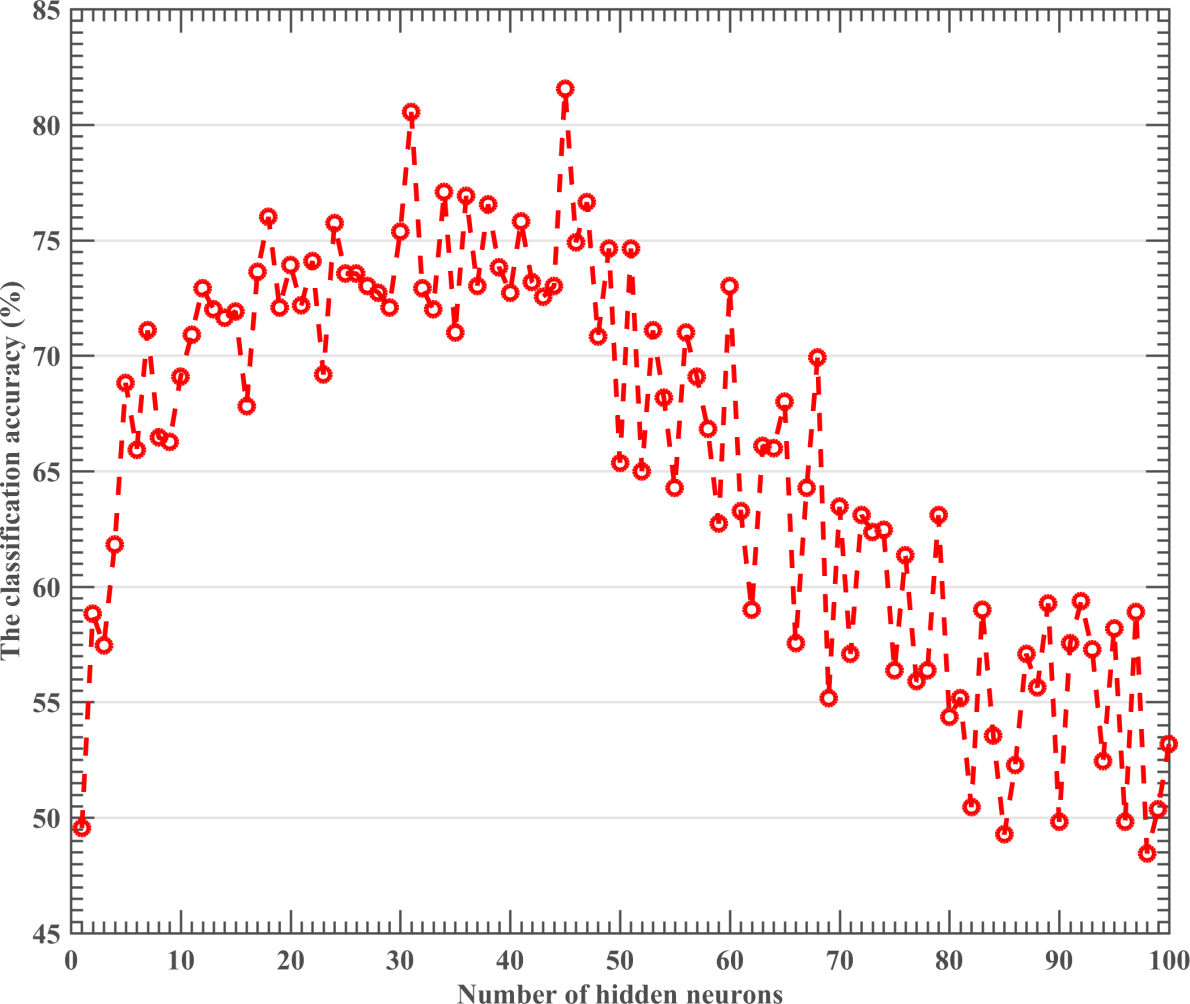
Classification accuracy of ELM versus the number of hidden neurons.

Statistical analysis showed that the coagulation, liver, and kidney indices possessed prognostic value and that 86.4% of the original grouped cases correctly classified according to Fisher discriminant analysis. For more accurate results, a machine learning method was developed. As the most important parameter in ELM was the number of hidden neurons, the relationship between the classification accuracy and the number of hidden neurons was first investigated. In this experiment, the number of hidden neurons was increased from 1 to 100 with steps of 1. Fig 3 shows the relationship between validation accuracy and the number of hidden neurons. As shown, the classification accuracy of ELM was sensitive to the number of hidden neurons. ELM achieved the best accuracy when the number of hidden neurons was equal to 45 and, thus, 45 hidden neurons were chosen to create the ELM in the following implementation. As for the activation function, the Sigmoid function was chosen as it produced the most stable and highest results in the experiment.

After the number of hidden neurons and activation function were determined, the ELM model on the combined feature space was evaluated, including the coagulation, liver, and kidney indices. Table 4 lists results obtained from the ELM model with its whole features. The ELM achieved 81.45% ACC, 0.6379MCC, 80.70% sensitivity, and 85.06% specificity.

**Table 4:** The detailed results obtained by ELM.

Fold	ACC	MCC	Sensitivity	Specificity
#1	0.9091	0.8101	0.8750	1.0000
#2	0.7000	0.5345	0.5714	1.0000
#3	1.0000	1.0000	1.0000	1.0000
#4	0.6000	0.1667	0.6667	0.5000
#5	0.6000	0.1021	0.5000	0.6250
#6	0.7273	0.4667	0.8000	0.6667
#7	1.0000	1.0000	1.0000	1.0000
#8	0.8000	0.6547	1.0000	0.7143
#9	0.9091	0.8281	0.8571	1.0000
#10	0.9000	0.8165	0.8000	1.0000
Avg.	0.8145	0.6379	0.8070	0.8506
Dev.	0.1516	0.3179	0.1790	0.2000

Avg. and Dev. means the average value and standard deviation of the 10-fold CV results.

### 5.5 Evaluation of ELM model

**Fig 4:**
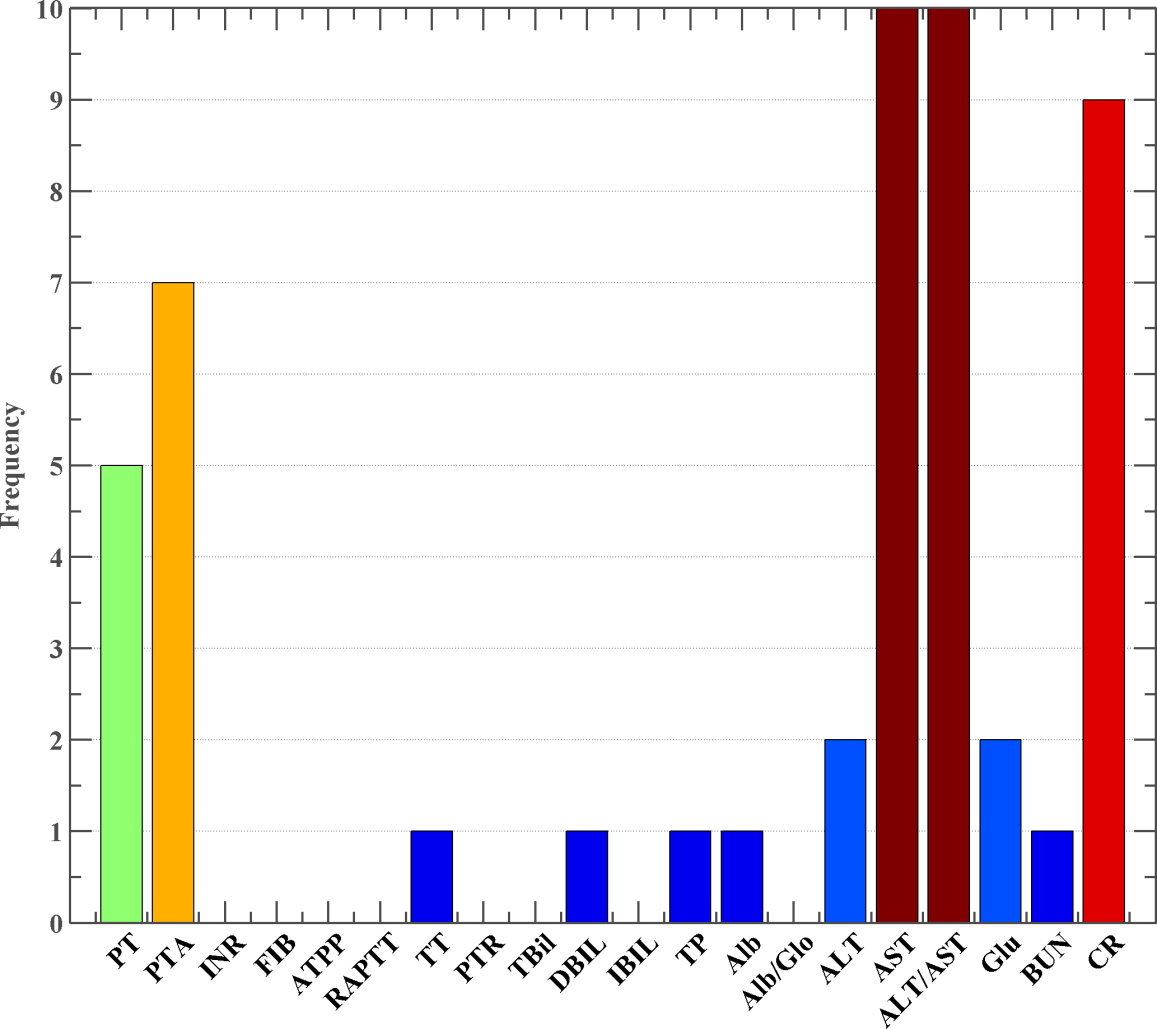
The frequency of selected features in 10-fold CV.

The performance of the ELM model was further evaluated by applying the proposed GWO-wrapped ELM model to the same data. Table 5 displays detailed results obtained by this proposed GWO-ELM model. The method produced excellent results, with 86.55% ACC, 0.7407MCC, 81.24% sensitivity, and 90.48% specificity. Also, the model identified the most significant feature subset on each fold data. In the obtained 10 feature subsets, five indices, including AST, AST/ALT, CR, PT, and PTA, appeared most frequently in these 10 feature subsets (Fig 4). Compared with the ELM with the whole feature set, GWO-ELM boosted results by 5.1, 10.28, 0.54, and 5.42% in terms of ACC, MCC, sensitivity, and specificity, respectively. This indicated that certain redundant and irrelevant features existed in the data. Additionally, it was interesting to note that the standard deviation obtained by model was much smaller than that of the ELM model with the whole features, which indicated that the GWO-ELM was much more stable on reduced feature space.

**Table 5:** The detailed results obtained by GWO-ELM.

Fold	Selected feature subset	ACC	MCC	Sensitivity	Specificity
#1	{AST,ALT/AST,CR,PT,PTA}	0.9000	0.8018	1.0000	0.7500
#2	{ALT,AST,ALT/AST,CR,PT}	0.7273	0.4485	0.7500	0.7143
#3	{AST,ALT/AST,PTA}	0.9000	0.7638	0.6667	1.0000
#4	{TP,AST,ALT/AST,Glu,CR,PTA,TT,PTR}	0.9000	0.8018	0.8571	1.0000
#5	{AST,ALT/AST,CR,PTA}	0.9091	0.8333	1.0000	0.8333
#6	{AST,ALT/AST,CR,PT}	0.9000	0.8018	0.7500	1.0000
#7	{AST,ALT/AST,CR,PTA}	0.9000	0.8165	0.8333	1.0000
#8	{DBIL,ALT,AST,ALT/AST,Glu,BUN,CR,PTA,PTR}	0.9000	0.8018	1.0000	0.7500
#9	{Alb,AST,ALT/AST,CR,PT,PTA}	0.8182	0.6708	0.6000	1.0000
#10	{AST,ALT/AST,CR,PT,PTR}	0.8000	0.6667	0.6667	1.0000

**Fig 5:**
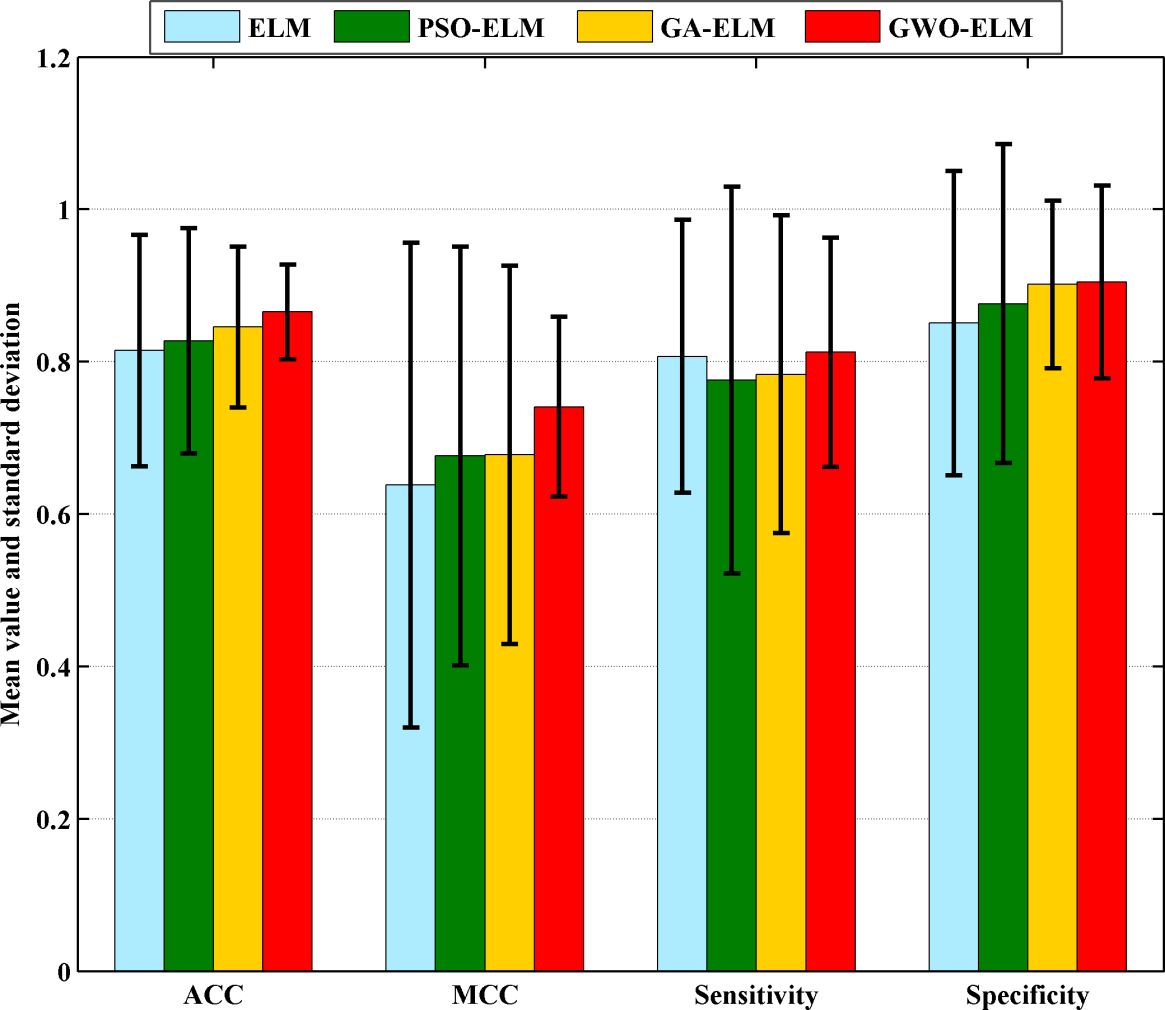
The classification performance obtained by the four methods in terms of ACC, MCC, sensitivity, and specificity.

With the purpose of verifying the effectiveness of the constructed GWO-ELM, the other metaheuristics-based ELM methods, including GA-ELM and PSO-SVM, and the original ELM were used for comparison. Fig 5 displays the mean ACC and standard deviations obtained by each method via a 10-fold CV. The GWO-ELM surpassed the original ELM, GA-ELM, and PSO-ELM in terms of the ACC, MCC, sensitivity, and specificity, which meant that the GWO-based feature-selection strategy helped ELM to further boost its classification performance. It was interesting to find that the GWO-ELM standard deviation was much smaller than the other three competitors in terms of most evaluation metrics, only exhibiting a slightly larger value than GA-ELM in terms of specificity. Hence, the ELM construct, based upon GWO, lead to more stable classification results. In short, the GWO not only helped increase the ELM classification results but also resulted in a more stable and robust classifier for prognosticating PQ poisoning. The comparison results indicated that GWO-ELM was the most stable and robust method for PQ poisoning prognosis, followed by GA-ELM, PSO-ELM, and ELM.

**Fig 6:**
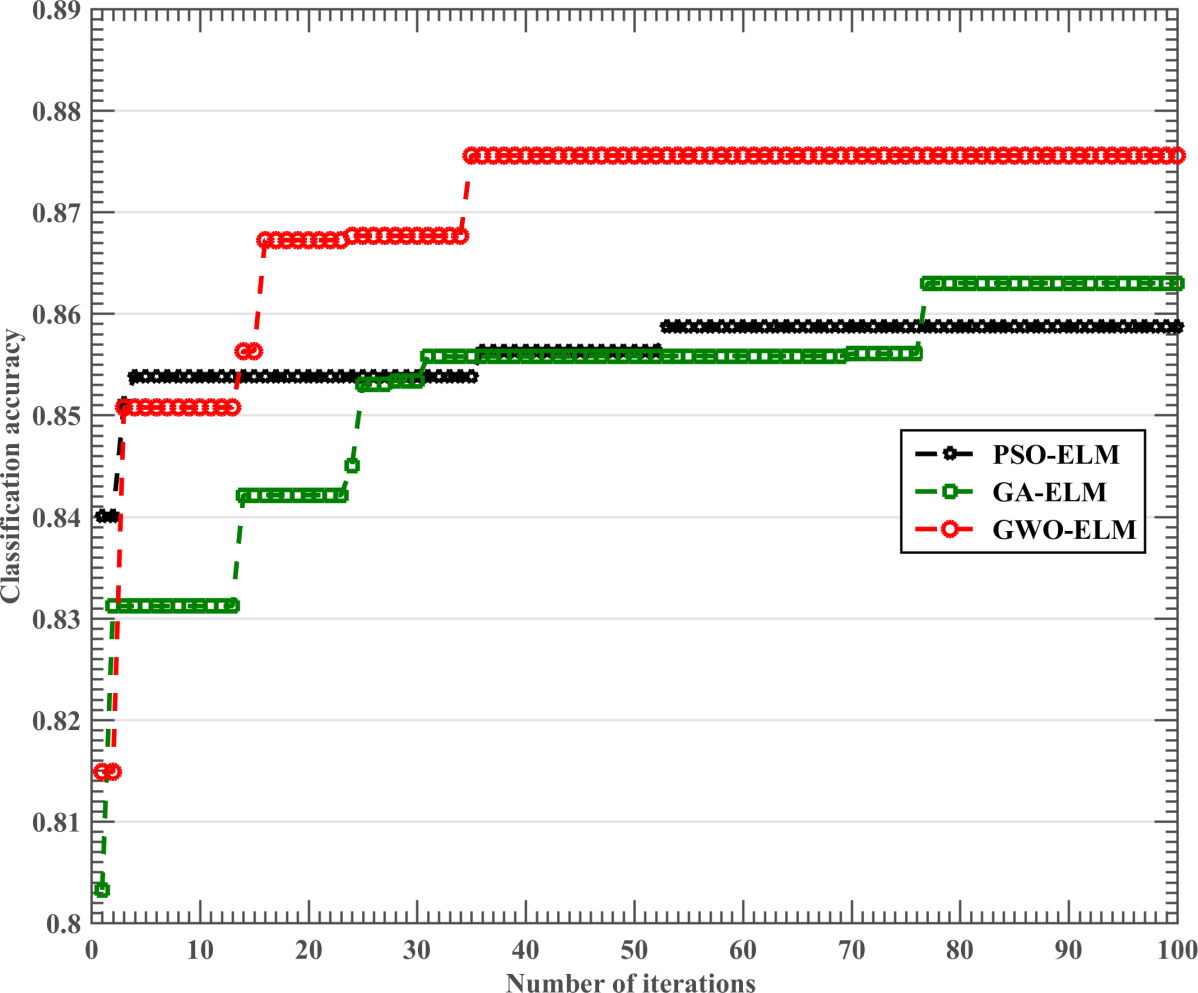
The mean result of the best fitness during the training stage in 10-fold CV procedure obtained by the three methods.

The optimization procedures of the metaheuristic optimization methods, including GWO-ELM, PSO-ELM and GA-ELM, were explored by recording the evolutionary process of the three methods (Fig 6). It was observed that the GWO strategy exhibited an obvious advantage over the other methods not only in terms of convergence rate but also quality of solution, and only 35 iterations were needed to achieve the best fitness. In contrast, it took PSO and GA 53 and 78 iterations, respectively, to reach to maximum fitness. Another special fact was that PSO showed a better convergence rate than GA, although its maximum fitness was smaller than GA-ELM, which meant that PSO possessed superior performance over GA in terms of the convergence rate. In short, GWO significantly improved ELM’s convergence rate and the solution quality as well.

Table 6 presents the frequenciesof the selected features of GA-ELM, PSO-ELM, and GWO-ELM within the 10-fold CV procedure. It was interesting to find that the three methods consistantly picked out five indices, including AST, AST/ALT, CR, PT, and PTA, which most frequently appeared in 10 feature subsets. Other than these five indices, GA-ELM also chose TBil and ALT indices >6 times out of 10, while PSO-ELM picked out DBIL and Glu indices >7 times out of 10. It was observed from the data that the frequency of AST, AST/ALT, CR, PT, and PTA selected by GWO-ELMwerehigher than the other two counterparts, while other indiceschosen by GWO-ELMwerelower than the other two counterparts. On one hand, this indicated that GWO possessed better capability for removing redundant and relevant features from the data. On the other hand, it also suggested that the GWO strategy picked out the most discriminative features from the dataset, better than the other two counterparts.

**Table 6:** Average frequencies of the selected features by the three methods.

Feature	Average selected frequencies		
	GA-ELM	PSO-ELM	GWO-ELM
PT	5	5	5
PTA	5	6	7
INR	0	2	0
FIB	1	3	0
ATPP	2	0	0
RAPTT	0	4	0
TT	2	3	1
PTR	1	2	0
TBil	6	1	0
DBIL	2	7	1
IBIL	3	0	0
TP	0	3	1
Alb	0	1	1
Alb/Glo	1	2	0
ALT	8	4	2
AST	7	8	10
ALT/AST	7	6	10
Glu	4	8	2
BUN	0	2	1
CR	8	6	9

**Fig 7:**
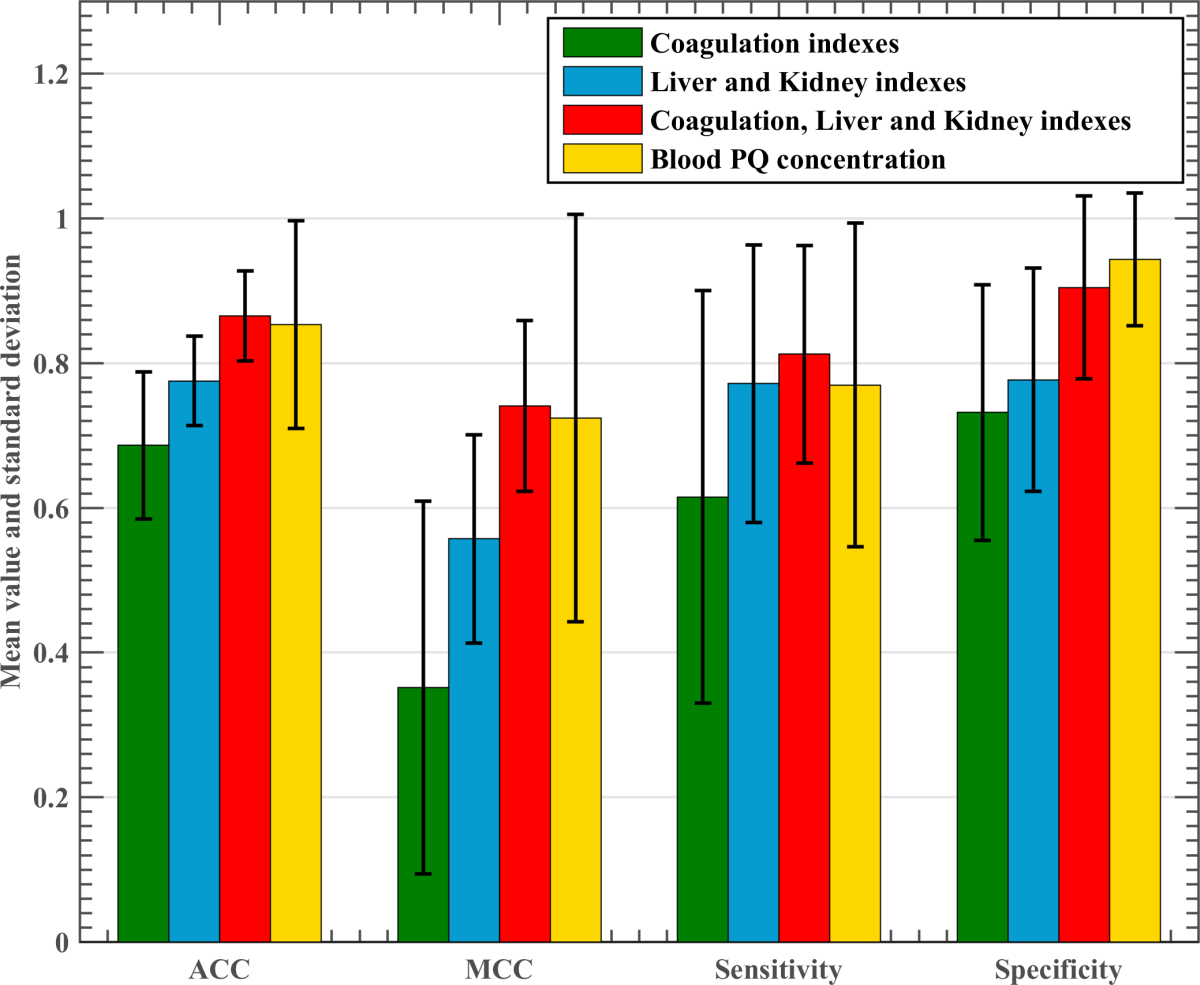
Classification performance obtained by the GWO-ELM method based on the four different indices in terms of ACC, MCC, sensitivity, and specificity.

The effectiveness of the fusion of coagulation, liver, and kidney indices were validated by testing the proposed GWO-ELM on different indices, including coagulation, liver and kidney indices, combinations of coagulation, liver, and kidney indices, and blood PQ concentration in terms of four different evaluation metrics. Fig 7 displays the detailed results of GWO-ELM results for different indices. GWO-ELM achieved 68.64% ACC, 0.3516 MCC, 61.50% sensitivity, and 73.19% specificity, based on coagulation indices; and 77.55% ACC, 0.5571 MCC, 61.50% sensitivity, and 73.19% specificity, based on liver and kidney indices. However, when the two kinds of indices were fused, they produced 86.55% ACC, 0.7407 MCC, 81.24% sensitivity, and 90.48% specificity, which was even higher than that obtained with blood PQ concentration, which produced 85.36% ACC, 0.7243 MCC, 77.00% sensitivity, and 94.33% specificity. Additionally, the standard deviation of GWO-ELM on the fused indices was found to be the smallest among the four kinds of indices. This indicated that the fused indices aided the GWO-ELM in offering more robust and stable prognostic results.

## 6. Discussion

Coagulation is the process in which liquid blood forms a blood clot by extrinsic or intrinsic pathways of coagulation. This process has many biochemical reactions and involves platelets, a series of proteases, and a coagulation factor, such as prothrombin, proconvertin, and antihemophilic factor B. When blood vessels are damaged or endothelium cells exposed, the coagulation function is instantly activated. If any proteases or coagulation factors are destroyed, the coagulation reactions will be in disorder and result in increased clotting time.

In this study, coagulation test indices were analyzed first and the results showed that, in the deceased group, PTwas increased and PTA decreased (*p*<0.05), while the other coagulation indices, such as APTT, showed no significant differences. This indicated that some of coagulation factors, including I (fibrinogen), II (prothrombin), V, VII, and X, were inhibited in PQ patients. PT is different from APTT, with the latter used to evaluate the intrinsic coagulation pathway and the former to measure the extrinsic coagulation pathway which involves those coagulation factors. Coagulation factors I, II, V, VII, and X are synthesized in liver and, therefore, liver function was damaged in PQ-poisoned patients.

Except for increased PT, ALT and AST were both increased in the deceasedgroup.ALT and AST are found in plasma and in various body tissues but are most common in the liver. Serum ALT, AST, and their ratio (AST/ALT) are the most commonly clinical indices for liver function. Therefore, by combining ALT and AST results, it was confirmed that liver function was more seriously damaged in the deceased group than the survival group. Similarly, increased concentrations of TBil, DBIL, IBIL, BUN, and CR indicated that kidney function was also seriously damaged in the deceased group.

Although, there were significant differences between the survival and deceased groups, the prognostic value of these tests was not attained according to statistical analysis. The prognostic values of these indices were evaluated using a new machine learning method, GWO-ELM, which was proposed here for the first time. The results showed that AST, AST/ALT, CR, PT, and PTA were the most important indices correlated with PQ-poisoning prognoses. These five indices were selected by the proposed method as they all showed statistical differences, which indicated that the proposed method was convincing. AST is distributed widely in various body tissues, such as liver, heart, skeletal muscle, kidneys, brain, and red blood cells; correspondingly, ALT is rich in liver. Therefore, AST was a more important prognostic value than ALT.

To date, PQ concentration has always been considered as the most relative, sensitive, and accurate index for identifying the degree of PQ toxicity. The prognostic value of blood routine examination and arterial blood gas tests in PQ poisoning were evaluated and neither of the two examinations reached the same sensitivity in prognosis of PQ poisoning as blood PQ concentration [8, 9]. However, in this study, when the indices of coagulation, liver, and kidney tests were combined, the results showed the sensitivity higher than that of blood PQ concentration. AsPQ isexcreted and blood PQ concentration decreases with time, indices of coagulation, liver, and kidney do not. In the last blood tests, coagulation, liver, and kidney tests showed that the statistical differences of indices of coagulation, liver, and kidney were almost the same as in the first tests upon hospital admission (Table 2). Therefore, coagulation, liver, and kidney tests exhibited important prognostic values and should be constantly monitored.

## 7. Conclusions

In this study, the predictive value of coagulation, liver, and kidney indices in prognosticating patients with PQ poisoning was investigated. Statistical analysis showed that PT, PTA, INR, TBil, DBIL, IBIL, ALT, AST, ALT/AST, BUN, and CR were highly correlated to PQ poisoning and showed statistical significance (*p*<0.05) in predicting the prognosis of PQ poisoning.

Accuracy of results in predicting the prognosis of PQ poisoning was obtained using a new machine learning technique, GWO-ELM, that was proposed here and able to pick out the most significant indices for evaluating PQ-poisoning prognoses. The maximal classification accuracy approached 90%, which was higher than that of blood-PQ concentration. Therefore, the combination of coagulation, liver and kidney indices was concluded here to be of significance for predicting the outcome of patients with PQ poisoning.

## Supporting information

S1 Dataset The file contains the first time tests of coagulation, liver, kidney indices, deceased group (1) and survival group (2).
(XLSX)
